# A de novo microdeletion involving *PAFAH1B* (*LIS1*) related to lissencephaly phenotype

**DOI:** 10.1016/j.dib.2015.07.017

**Published:** 2015-07-23

**Authors:** Keiko Shimojima, Akihisa Okumura, Toshiyuki Yamamoto

**Affiliations:** a Precursory Research for Embryonic Science and Technology (PRESTO), Japan Science and Technology Agency (JST), Kawaguchi, Japan; b Tokyo Women׳s Medical University Institute for Integrated Medical Sciences (TIIMS), Tokyo, Japan; c Department of Pediatrics, Juntendo University, Tokyo, Japan; d Department of Pediatrics, Aichi Medical University, Nagakute, Japan

## Abstract

Lissencephaly is a type of the congenital malformation of the brain. Due to the impairments of neuronal migration, patients show absence of brain convolution manifesting smooth brain surfaces. One of the human genes responsible for lissencephaly is the platelet-activating factor acetylhydrolase 1b gene (*PAFAH1B*; also known as *LIS1*) located on 17p13.3. Patients with heterozygous deletion of this chromosomal region exhibit lissencephaly. Recently, we encountered a male patient who showed typical lissencephaly. Using a microarray analysis, we identified a 1.3 Mb submicroscopic deletion in 17p13.3. This deletion included *PAFAH1B*. Both of the parents showed no deletion in this region. Therefore, this was determined to be derived from de novo origin. After obtaining the written informed consent, skin fibroblasts were provided from this patient and disease-specific induced pluripotent stem (iPS) cells were generated and used for medical research (Shimojima K, Okumura A, Hayashi M, Kondo T, Inoue H, and Yamamoto T. CHCHD2 is down-regulated in neuronal cells differentiated from iPS cells derived from patients with lissencephaly. Genomics, in press).

Specifications table

**Table:** 

Subject area	Biology
More specific subject area	Genomics
Type of data	Patient descriptions and resulted data analyzed by microarray-based comparative genomic hybridization (CGH) analysis and fluorescence in-situ hybridization (FISH) analysis
How data was acquired	Using Agilent SureScan Microarray Scanner, Agilent Genomic Workbench software, and Leica CTR6000
Data format	Descriptions of a patient and captured figures by equipments
Experimental factors	Clinical information and biological samples from a patient who exhibited lissencephaly
Experimental features	Using microarray-based comparative genomic hybridization (CGH) analysis and fluorescence in-situ hybridization (FISH) analysis, genomic copy number aberration was analyzed in a patient who exhibited lissencephaly.
Data source location	Tokyo Women׳s Medical University Institute for Integrated Medical Sciences (TIIMS), Tokyo, Japan
Data accessibility	These data are with this article.

Value of the data

• A 3-year-old boy showed epileptic spasms at 4 months.
• Brain magnetic resonance imaging (MRI) demonstrated lissencephaly in this patient.
• Microarray-based comparative genomic hybridization (CGH) analysis identified a 1.3 Mb deletion in 17p13.3 region, in which *PAFAH1B* is located.
• Fluorescence in-situ hybridization (FISH) analysis confirmed a deletion in the patient but not in his parents, indicating *de novo* origin.

## Data, experimental design, materials and methods

### Clinical information

A 3-year-old boy was born after 40 weeks of gestation with a birth weight of 3270 g. He was the first product of non-consanguineous healthy parents. There was no family history of neurological disorders. His perinatal history was unremarkable, whereas a paucity of social smile and eye following was noticed at his 3-month health check-up.

**Fig. 1:**
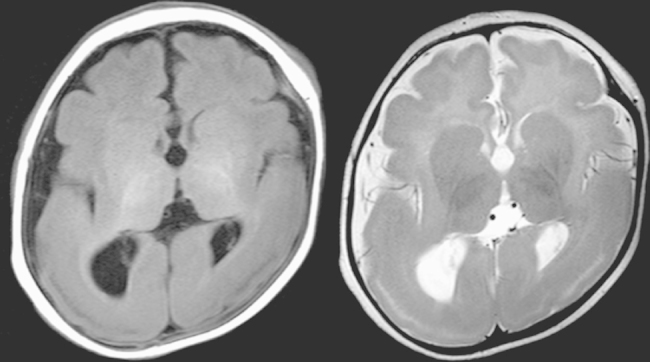
Brain magnetic resonance images of the patient examined at 5 months. T1- (left) and T2- (right) weighted axial images show volume loss and hypomorphic convolution of the brain cortex.

He had had clusters of epileptic spasms after 4 months of age. Within a week, the frequency of epileptic spasms increased to several clusters per day and he was admitted to our hospital. On admission, neurological examinations showed mild hypotonia of the trunk and the extremities with normal reflexes. There was no dysmorphic features. Electroencephalogram (EEG) showed hypsarrhythmia associated with high voltage theta activities, predominant in the bilateral temporo–parieto–occipital regions. Brain magnetic resonance imaging (MRI) demonstrated lissencephaly with agyria in the parieto–occipital regions and pachygyria in the fronto–central regions (Fig. 1).

He was treated with ACTH therapy. Although the epileptic spasms were markedly reduced by ACTH, complete seizure cessation was not achieved. Valproate and clobazam were administered after ACTH therapy and his seizures were controlled transiently. However, a recurrence of epileptic spasms occurred 2 weeks later. ACTH therapy was performed again at 7 months of age and epileptic spasms were completely controlled. After 12 months of age, he had seizures associated with asymmetric fencing-like posture lasting for 10 s. Ictal EEG revealed focal rhythmic fast activities in the bilateral frontal regions, thus his seizures were judged to be focal. Valproate, clobazam, topiramate, and levetiracetam were administered, but these drugs were not effective. He had 30–50 focal seizures every day. In addition, epileptic spasms also recurred after 18 months of age. At 21 months of age, ACTH therapy was again performed in combination with the administration of high dose phenobarbital. Epileptic spasms were eliminated and focal seizures were markedly reduced to 2–3 seizures per week.

At the last follow-up at 44 months of age, he had a few focal seizures per week and no epileptic spasms under treatment with phenobarbital and topiramate. However, his psychomotor development was severely delayed. No head control had achieved and verbal communication remained impossible.

Conventional chromosomal analysis showed a normal male karyotype of 46,XY.

### Molecular cytogenetic diagnosis

**Fig. 2:**
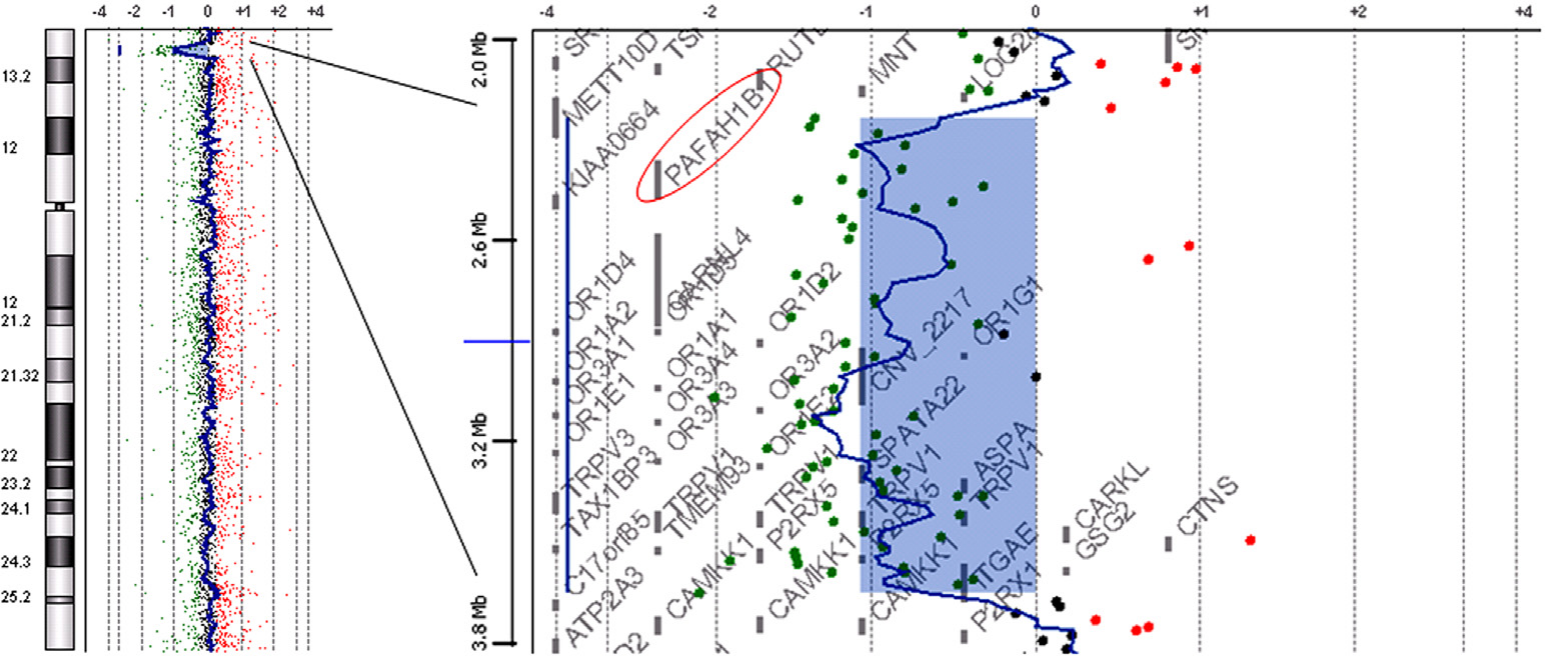
The result of chromosomal microarray testing. Schematic representation of chromosome 17 visualized by Chromosome View of Agilent Workbench (left; Agilent Technologies) shows an aberration at 17p13.2-p13.3, which is expanded by Gene View (right). *PAFAH1B* (*LIS1*) is included in the aberration region (a red circle).

Chromosomal microarray testing using Agilent Human Genome microarray 60 K (Agilent Technologies, Santa Clara, CA) was performed according to the method described previously [1], [2]. Using QIAamp DNA extraction kit (Qiagen, Hilden, Germany), genomic DNA was extracted from blood samples obtained with written informed consent, following approval by the ethical committee of our institution. The result showed an aberration with a 1.3 Mb width and a mean log_2_ ratio of –1.086387, indicating arr 17p13.3p13.2(2,371,080–3,686,008)×1 (Fig. 2), according to build19.

**Fig. 3:**
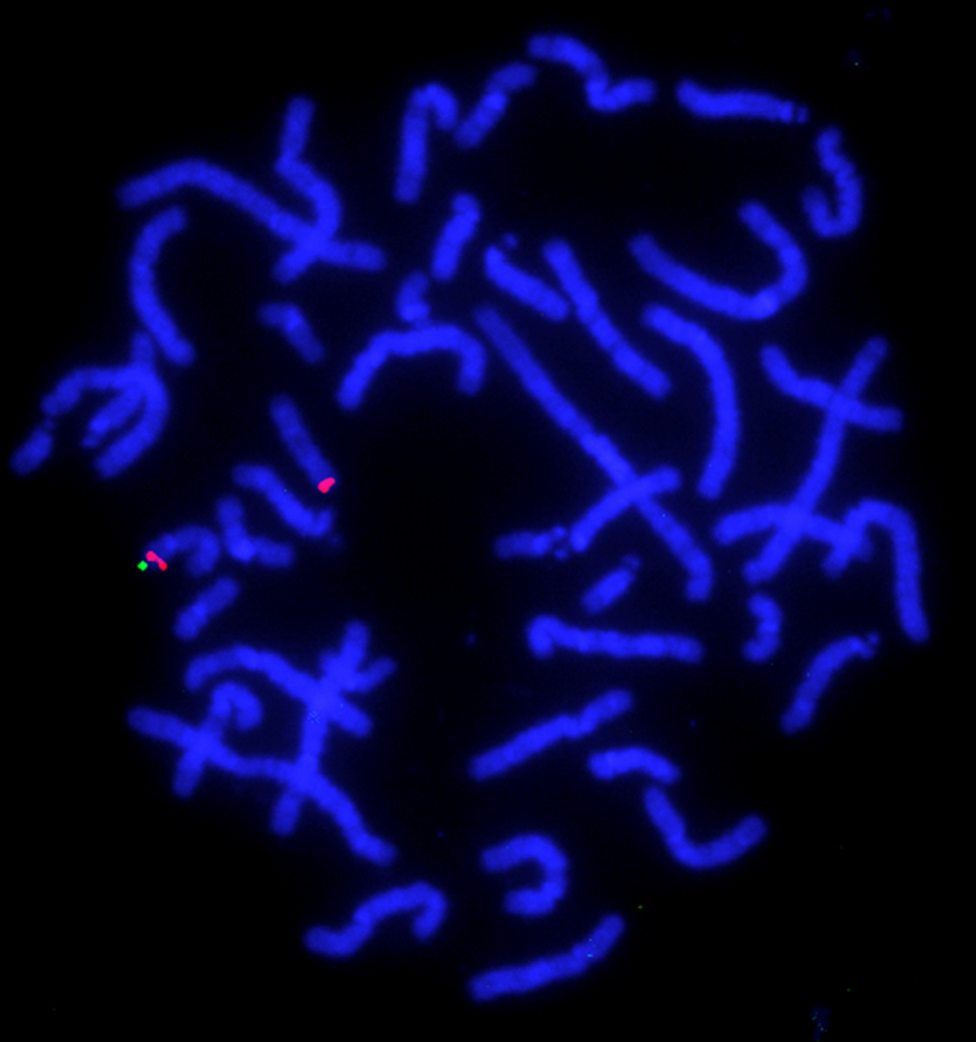
The result of FISH. A single green signal labeled for CTD-2576K4 indicates a deletion of this region. Two red signals labeled for RP11-1D5 are the marker of chromosome 17.

Then, fluorescence in-situ hybridization (FISH) analysis was performed to confirm the result of chromosomal microarray testing, using human bacterial artificial chromosomes (BAC) as the probes; CTD-2576K4 (chr17p13.3:2,492,176–2,643,505) as a target and RP11-1D5 (chr17p13.1:7,918,567–8,082,208) as a marker referring build19, which were selected from the UCSC genome browser (http://www.gwenome.ucsc.edu)(GRCh37/hg19). Metaphase spreads prepared from peripheral blood lymphocytes by using standard methods were used as described previously [1], [2]. Finally, a single signal of the target was identified, indicating a heterozygous deletion in this patient (Fig. 3). Because both of the parents did not show any signal deletion, de novo origin was determined in this case.

Induced pluripotent stem (iPS) cells were generated from this patient, and used for the medical research [3].
